# The HSV-1 ICP4 Transcriptional Auto-Repression Circuit Functions as a Transcriptional “Accelerator” Circuit

**DOI:** 10.3389/fcimb.2020.00265

**Published:** 2020-06-24

**Authors:** Sonali Chaturvedi, Ruth Engel, Leor Weinberger

**Affiliations:** ^1^Gladstone Institute of Virology and Immunology, University of California, San Francisco, San Francisco, CA, United States; ^2^Gladstone Center for Cell Circuitry, University of California, San Francisco, San Francisco, CA, United States; ^3^Department of Biochemistry and Biophysics, University of California, San Francisco, San Francisco, CA, United States; ^4^Pharmaceutical Chemistry, University of California, San Francisco, San Francisco, CA, United States

**Keywords:** herpesvirus, single-cell time-lapse imaging, fitness advantage, accelerator circuit, autoregulation, transcriptional feedback, HSV-1

## Abstract

Herpes simplex virus-1 (HSV-1) is a significant human pathogen. Upon infection, HSV-1 expresses its immediate early (IE) genes, and the IE transcription factor ICP4 (infectious cell protein-4) plays a pivotal role in initiating the downstream gene-expression cascade. Using live-cell time-lapse fluorescence microscopy, flow cytometry, qPCR, and chromatin immunoprecipitation, we quantitatively monitored the expression of ICP4 in individual cells after infection. We find that extrinsic stimuli can accelerate ICP4 kinetics without increasing ICP4 protein or mRNA levels. The accelerated ICP4 kinetics—despite unchanged steady-state ICP4 protein or mRNA level—correlate with increased HSV-1 replicative fitness. Hence, the kinetics of ICP4 functionally mirror the kinetics of the human herpesvirus cytomegalovirus IE2 “accelerator” circuit, indicating that IE accelerator circuitry is shared among the alpha and beta herpesviruses. We speculate that this circuit motif is a common evolutionary countermeasure to throttle IE expression and thereby minimize the inherent cytotoxicity of these obligate viral transactivators.

## Introduction

As one of the largest families of DNA viruses, Herpesviridae infections are prevalent among the world's population. The herpesvirus family is composed of three subfamilies: α, β, and γ. Viruses within the three subfamilies share little genetic similarity, but all infect substantial fractions of the human population, and viruses from each subfamily are associated with significant morbidity and mortality. For example, herpes simplex virus-1 (HSV-1), a member of the α-subfamily, infects ~70% world population (Pellett, 2006). HSV-1 is the leading cause of infectious blindness and a major cause of morbidity and mortality for organ transplant recipients and other immunocompromised populations. Human cytomegalovirus (HCMV) belongs to the β-subfamily and is a leading cause of birth defects and transplant failure in both bone-marrow and solid-organ transplants (Pellett, 2006).

**Figure 1:**
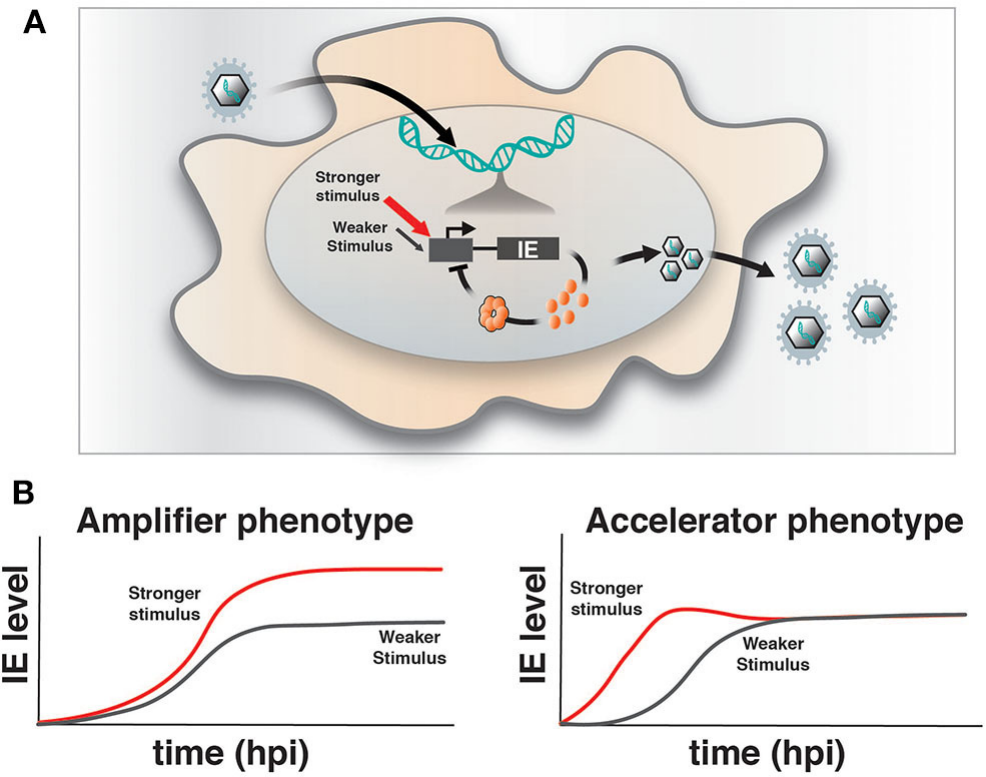
Herpesvirus immediate-early feedback circuitry and the accelerator-circuit assay. **(A)** Schematic of immediate early (IE) circuit in herpesviruses. IE promoter activity is responsive to the cell's transcriptional environment and is modulated by stronger (red arrow) or weaker (gray arrow) stimuli (input) that can generate differences in transcriptional output. IE gene products, such as the HCMV IE2 and HSV-1 ICP4 “master” transcriptional regulator proteins, transactivate downstream viral gene expression, but can also bind to their own promoters in an autoregulatory (i.e., negative feedback) fashion. **(B)** Overview of the assay to identify accelerator-like circuitry based on the transcriptional input–output relationship [based on (Teng et al., 2012)]. The assay is based on discrimination between alternate kinetic expression profiles (i.e., phenotypes) at the single-cell level. Left: Upon increasing input stimulus strength, amplifier circuitries generate increased output levels of steady-state expression (red) compared to weaker input stimuli (gray), (i.e., the amplifier phenotype). Right: In contrast, accelerator circuitries generate faster kinetics of expression (red) when input stimulus strength is increased, but these trajectories do not generate an increase in steady-state expression level compared to weaker input stimuli (gray), (i.e., the accelerator phenotype).

Upon infection of a permissive cell, α and β herpesviruses initiate their replicative programs and must simultaneously counteract silencing by innate cellular responses (Everett et al., 2003; Paludan et al., 2011). The immediate early (IE) viral genes play a crucial role in counteracting silencing and activating expression of downstream viral gene cascades to drive replication and propagation of the virus. However, a number of obligate IE genes are cytotoxic, such that their expression is stochastically silenced during infection of non-permissive cells (Cohen et al., 2020) and is tightly regulated in permissive cells. For example, we previously reported that HCMV encodes a transcriptional “accelerator” circuit that autoregulates expression of its master transcriptional regulator IE2 to prevent IE protein levels from reaching a cytotoxic threshold (Teng et al., 2012; Vardi et al., 2018). This accelerator circuit is in essence a highly non-linear, self-cooperative transcriptional negative-feedback circuit wherein IE2 protein binds as a high-order homomultimer to a specific site on its own promoter to repress transcription (Figure 1A). Consequently, a homeostatic level of IE2 is maintained irrespective of promotor strength, but IE2 kinetics can be modulated by promoter activation, [i.e., “acceleration-without-amplification” (Figure 1B)]. This phenotype represents a peculiar kinetic profile distinct from the more typical and expected “amplifier” profile and can typically be generated only by highly cooperative negative-feedback circuits (i.e., a Hill coefficient >1), a circuitry that appears in a broad array of biological systems (Gao and Stock, 2018). In HCMV, this circuitry enables accelerated IE expression in response to stimuli and confers a replicative fitness advantage to the virus by maintaining IE2 below the cytotoxic threshold (Teng et al., 2012). To determine whether acceleration without amplification is a conserved regulatory mechanism across herpesviruses, we examined if IE genes in HSV-1 exhibit similar property as IE2 in HCMV. HSV-1 encodes a similar accelerator circuitry to control expression of its IE gene ICP4, and the ICP4 acceleration-without-amplification phenotype similarly confers replicative fitness advantage to HSV-1.

HSV-1, upon infecting a permissive cell, expresses five IE genes that initiate the process of virus replication. One of the IE gene products, ICP4, is a 175-kDa phosphoprotein consisting of N-terminus activation domain, DNA-binding domain, linker domain, and a c-terminal activation domain (Honess and Roizman, 1975). ICP4 serves as the obligate transactivator of downstream viral gene expression and HSV-1's lytic cycle (DeLuca and Schaffer, 1985). However, like HCMV IE2, ICP4 is cytotoxic when overexpressed (Krisky et al., 1998) and represses its own expression by interacting with a specific 15-bp sequence in the *ICP4* promoter (Godowski and Knipe, 1986; Gu et al., 1993). However, despite these similarities to HCMV IE2, the mechanism and kinetics of ICP4 autorepression were undetermined.

## Materials and Methods

### Cells, Virus, Replication Kinetics

ARPE-19 and MRC5 cells were obtained from ATCC. The clinical strain of HSV-1 (17syn + ICP4-YFP) (Everett et al., 2003) was passaged from a clinical isolate (Brown et al., 1973) and kindly provided by Roger Everett, MRC Virology Unit, Glasgow, Scotland. Imaging was performed as described previously (Teng et al., 2012). Briefly, ARPE-19 cells were passaged onto a glass-bottom plate (Thermo Fisher Scientific) and grown to confluency to hold cells in G0. Cells were synchronously infected on ice for 30 min with HSV-1 strain 17syn + ICP4-YFP virus at MOI 1.0. Live cells were imaged with a 20× oil objective on a spinning disk confocal microscope (Olympus DSU) equipped with a 37°C, humidified 5% CO_2_ live-cell chamber. Image collection began when the YFP signal was first detected, and frames were captured every 10 min for 16–24 h with an exposure time between 200 and 800 ms (please see Supplementary Movie for a representative video of single-cell imaging of ICP4-YFP in ARPE-19 cells synchronously infected with HSV-1 strain 17syn + ICP4-YFP virus at MOI 1.0). Single-cell tracking and segmentation were performed with custom-written code in MatLab (MathWorks) as previously described (Weinberger et al., 2008). Replication kinetics of the virus were monitored at an early stage of infection in three biological replicates by infecting ARPE-19 cells with HSV-1-ICP4-YFP virus [MOI = 0.05] pretreated 24 h with HMBA (5 mM) or DMSO for three biological replicates in a 48-well plate. Cells were harvested by trypsinization at various time points post infection (0.5, 2, 8, 16, and 24 h), subjected to multiple freeze-thaws, and centrifuged, and the supernatant was used to calculate the virus titer by TCID-50 assay on MRC5 cells, as described previously (Nevels et al., 2004; Saykally et al., 2017). Titering performed in parallel on Vero cells showed almost identical trends and correlated well with ARPE and MRC5 titering but scaled by a constant value offset (i.e., quantitative, but no qualitative, titer differences were observed between ARPE, MRC5, and Vero).

### Flow Cytometry, RNA Extraction, Reverse Transcription, ChIP, and qPCR

For flow cytometry experiments, cells pretreated with HMBA or DMSO for 24 h followed by synchronized infection with HSV-1 (strain 17syn+ ICP4-YFP) [MOI = 1.0] were harvested at 5, 9, and 13 h post infection from three biological replicates and assayed for YFP by flow cytometry on LSRFortessa (BD Biosciences). ChIP was performed using protocol described previously (Silva et al., 2012) using antibody against YFP from cells pretreated with HMBA or DMSO for 24 h followed by infection with HSV-1 (strain 17syn+ICP4-YFP) [MOI = 1.0] using sequence-specific primers (ICP4 promoter forward: CGCATGGCATCTCATTACCG, ICP4 promoter reverse: TAGCATGCGGAACGGAAGC; GAPDH forward: TTCGACAGTCAGCCGCATCTT, GAPDH reverse: CAGGCGCCCAATACGACCAAA). For RNA extraction followed by qPCR, cells were pretreated with HMBA or DMSO for 24 h followed by infection with HSV-1 (strain 17syn+ ICP4-YFP) [MOI = 0.05], harvested 5, 9, 13, and 17 h post infection from three biological replicates, and reverse-transcription qPCR was performed as described previously (Vardi et al., 2018). Briefly, total RNA was extracted from cells using an RNeasy RNA Isolation kit (catalog no.: 74104, Qiagen) and RNA transcripts were made using QuantiTet Reverse Transcription Kit (catalog no.: 205311, Qiagen) according to the manufacturer's protocol. Reverse-transcribed cDNA samples were assayed by qPCR on a 7900HT Fast Real-Time PCR System (catalog no.: 4329003, Thermo Fisher Scientific) using Fast SYBR Green Master Mix (catalog no.: 4385612, Applied Biosystems) using sequence-specific primers (ICP4 mRNA forward: GCGTCGTCGAGGTCGT, ICP4 mRNA reverse: CGCGGAGACGGAGGAG). Relative mRNA level of ICP4 expression was quantified using peptidylprolyl isomerase A (PP1A) as a reference gene.

## Results and Discussion

Using time-lapse fluorescence microscopy, we followed ICP4 expression kinetics after infecting ARPE-19 cells with a previously characterized 17syn + HSV-1 encoding an ICP4-YFP fusion protein (Everett et al., 2003). ICP4 kinetics were quantified in individual cells using the imaging approach we developed previously (Teng et al., 2012; Vardi et al., 2018) in the presence or absence of hexamethylene bisacetamide (HMBA), an established transactivator of IE promoter expression (McFarlane et al., 1992).

**Figure 2:**
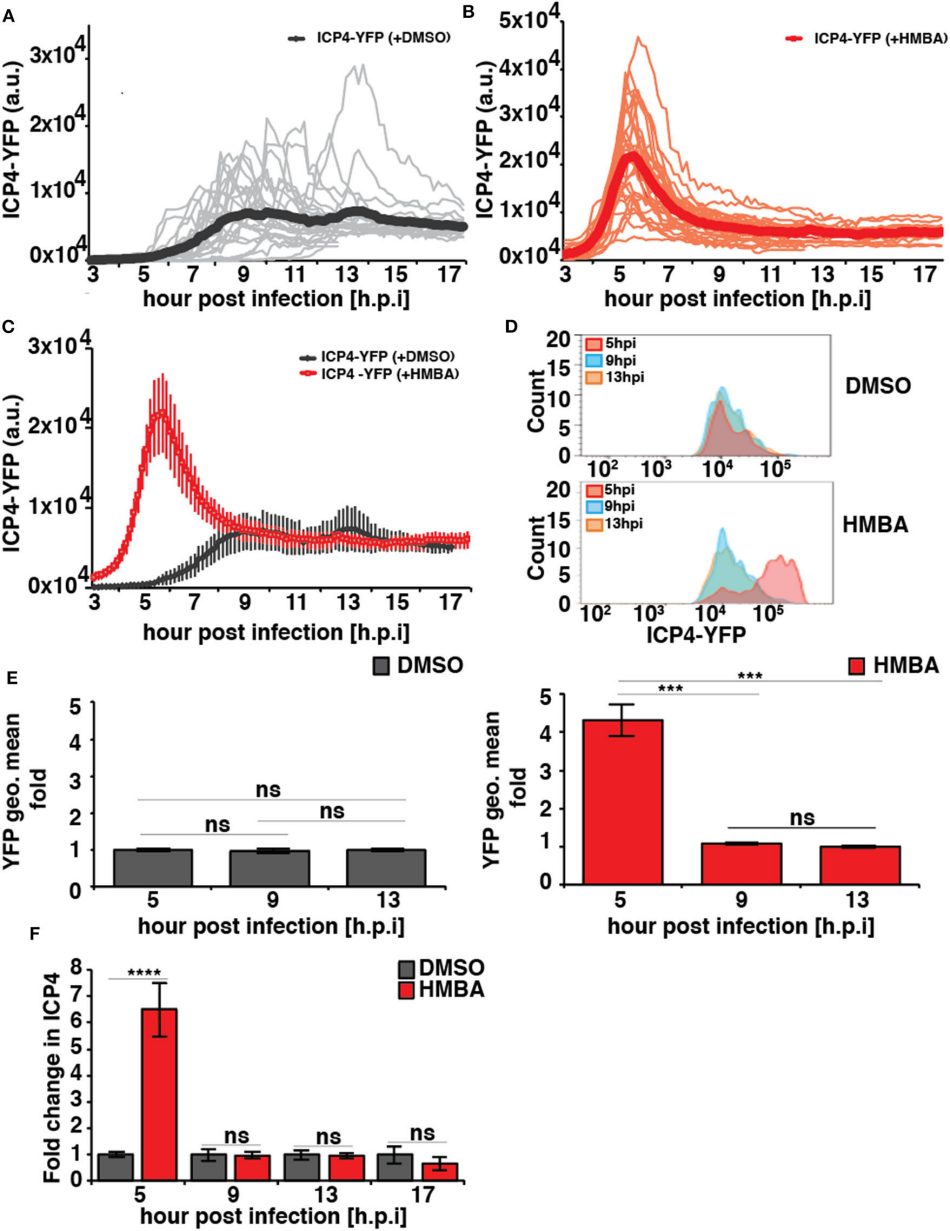
HSV-1 ICP4 single-cell, protein, and mRNA kinetics indicate the presence of an IE accelerator circuit. **(A,B)** Single-cell time-lapse microscopy traces of ICP4-YFP in ARPE-19 cells after infection with HSV-1 (strain 17syn + ICP4-YFP) at MOI = 1.0 in the presence of DMSO **(A)** or 5mM HMBA **(B)**. **(C)** Overlay of the mean ICP4-YFP expression in the presence of DMSO and HMBA. **(D)** Flow cytometry analysis of ARPE-19 cells infected with HSV-1 (strain 17syn + ICP4-YFP) in the presence of DMSO (top) or HMBA (bottom). **(E)** Mean and standard deviation of ICP4-YFP geometric mean of three biological replicates (13 hpi value normalized to one) from the flow cytometry data for different time points post infection (5, 9, and 13 h). **(F)** Quantification of ICP4 transcripts. ARPE-19 cells were infected with HSV-1 (strain 17syn + ICP4-YFP) at MOI of 0.05 in the presence of DMSO or HMBA, cells were harvested in triplicate at various time points, total RNA was extracted and reverse transcribed, and qPCR was performed to quantify ICP4 transcripts (ICP4 mRNA for DMSO sample was normalized to one). (*p*-value < 0.05 was considered statistically significant: *** < 0.001, **** < 0.0001, two-tailed *t* test).

In the absence of HMBA, ICP4-YFP kinetics in each cell followed stereotyped dynamics similar to HCMV IE2 kinetics, where the level of YFP monotonically rose to a peak at ~8 h post infection, generated a small overshoot, and then settled at a steady-state level (Figure 2A). In the presence of HMBA, ICP4-YFP levels increased far more rapidly and generated a much larger overshoot, but at ~9 h ICP4 levels in each cell reached a steady state that was indistinguishable from ICP4 levels in the absence of HMBA (Figure 2B). Overlaying the ICP4 traces in both conditions revealed that ICP4-YFP expression kinetics exhibit a strong acceleration-without-amplification phenotype in response to HMBA activation (Figure 2C). The single-cell imaging results were confirmed by flow cytometry analysis (Figures 2D,E). Flow cytometry analysis showed that HMBA induced a ~4-fold increase in ICP4-YFP intensity at 5 h with no significant difference in ICP4-YFP levels at 9 h post infection. The flow cytometry and imaging data general fall within comparable ranges, and the larger average fold increase in ICP4-YFP intensity measured by flow cytometry at 5 h compared to single-cell imaging at 5 h could be due to the smaller subset of cells analyzed by imaging or technical differences in detection methods.

To confirm that HMBA accelerated ICP4 transcription, ICP4 mRNA levels were assayed by RT-qPCR and a similar acceleration-without-amplification effect was observed at the mRNA level (Figure 2F). In agreement with protein level imaging and flow data, HMBA increased ICP4 mRNA levels at 5 h post infection by ~6.5-fold compared to DMSO controls and compared to 9 h post-infection, an increase that is slightly larger than imaging and flow data but within the ranges measured by these protein-level analyses. These differences in the peak level at 5 h may reflect differences between ICP4 protein and mRNA turnover rates.

**Figure 3:**
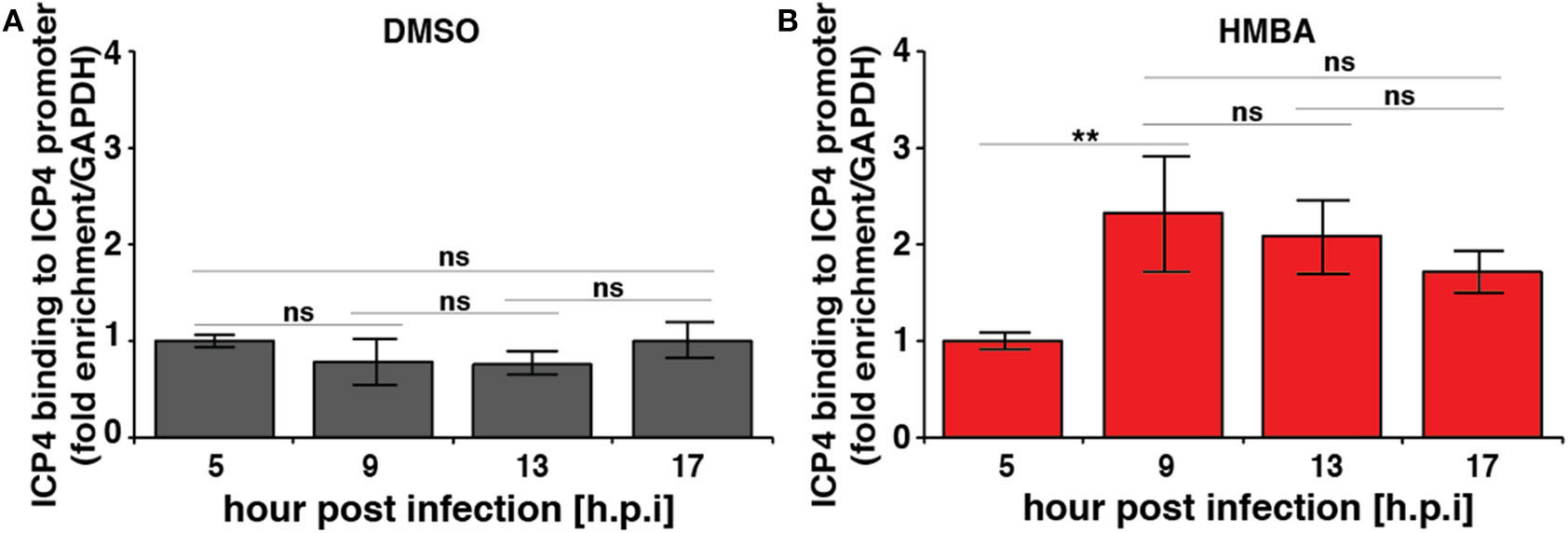
Chromatin immunoprecipitation (ChIP) analysis shows increased ICP4 binding to its own promoter coincident with decrease in ICP4 levels. ARPE-19 cells pretreated with DMSO **(A)** or HMBA **(B)** were infected with HSV-1 (strain 17syn + ICP4-YFP) [MOI = 1] followed by ChIP analysis of ICP4 protein and quantification of enriched DNA using qPCR primers specific for ICP4 promoter. Values were normalized to GAPDH to determine fold enrichment (*p* < 0.05 was considered statistically significant: ** < 0.01).

Computational modeling (Teng et al., 2012) indicates that for the acceleration-without-amplification phenotype to be achieved, the IE protein must negatively auto-regulate its promoter and theory predicts that negative-feedback strength must *increase* to mediate the reduction in expression levels and maintain unchanged steady-state levels. To test this hypothesis, we performed chromatin immunoprecipitation (ChIP) analysis to assay ICP4 the kinetics of binding to its own promoter over time in the presence and absence of HMBA stimulation (Figure 3). As predicted, ChIP results indicate that ICP4 binding to its own promoter is indeed significantly increased at 9 h post infection in the presence of HMBA, coincident with the decrease in ICP4 protein and mRNA levels observed (Figures 2, 3).

**Figure 4:**
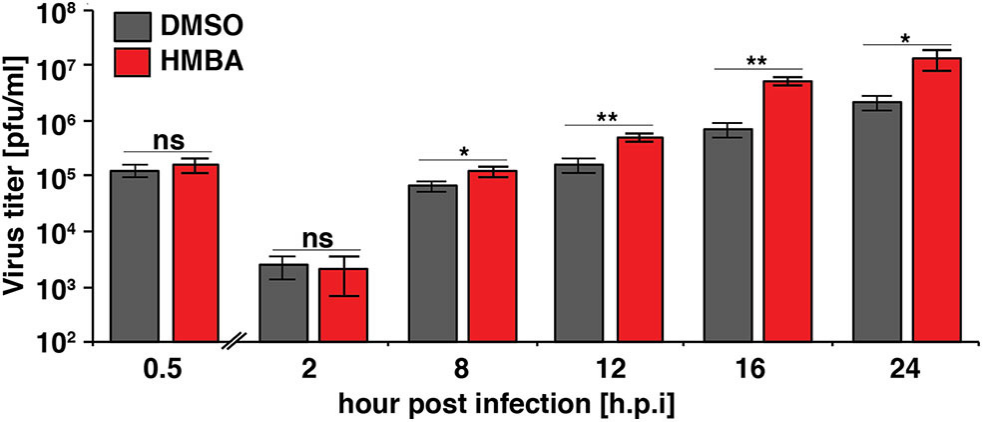
The ICP4 accelerator effect confers enhanced replicative fitness. ARPE-19 cells were infected with HSV-1 (strain 17syn + ICP4-YFP) at MOI of 0.05 in the presence of DMSO or HMBA before cells were harvested in triplicate at various time points (0.5–24 h) post-infection and virus titer analyzed on MRC5 cells. Fold change of DMSO to HMBA samples are shown (*p* < 0.05 was considered statistically significant: * < 0.05 and ** < 0.01).

We next examined the replication kinetics of HSV-1 in the presence and absence of HMBA to determine the effect of the acceleration-without-amplification phenotype on replicative fitness (Figure 4). Replication kinetics were calculated based on virus titer in cells infected with HSV-1 ICP4-YFP at MOI = 0.05 of (17syn+) in the presence of HMBA or DMSO for 0.5, 2, 8, 12, 16, and 24 h post infection (hpi). Similar to HCMV, acceleration of HSV-1 replication by HMBA treatment increased viral replicative fitness by ~4-fold.

Comparing the single-cell ICP4 trajectories to the virus infectious output (Figure 4), it is striking to note that although HMBA induced ICP4 accumulation between 5 and 8 hpi, HMBA had little effect on virus output at 8 hpi. In contrast, HMBA had little effect on ICP4 level at 12 and 16 h, but HSV-1 titers were significantly increased at those time points. Hence, despite identical levels of ICP4 (at 9 hpi and later), HSV-1 viral output is significantly higher, and the accelerated ICP4 expression kinetics early in infection correlate with this replicative fitness advantage for HSV-1.

Theoretical predictions (Teng et al., 2012) indicate that the accelerator phenotype requires binding of the IE protein to its promoter as a high-order homomultimer, likely consisting of five or more monomers. The results herein argue that the HSV-1 ICP4 auto-repression circuit is highly non-linear and self-cooperative—the criteria required for the accelerator circuit motif—and predicts that ICP4 likely binds to its promoter as a high-order homomultimer. In HCMV, ablation of the IE2 accelerator circuit drives IE2 levels above a cytotoxic threshold and leads to >2-log reductions in HCMV titers (Teng et al., 2012). Subsequent genetic studies are necessary to determine if ablation of the HSV-1 ICP4 accelerator circuitry would have a similar effect.

Overall, these data demonstrate that ICP4, which regulates the expression of downstream genes in HSV-1, encodes an accelerator circuit, which may play an important role in viral replication. Because overexpression of some IE genes can lead to cytotoxicity, the accelerator circuits that control IE2 and ICP4 expression may be examples of convergent evolution arriving at a shared mechanism to maintain IE2 and ICP4 levels below a cytotoxic threshold, in HCMV and HSV-1, respectively. Furthermore, the presence of this accelerator circuit motif in both α and β herpesviruses suggests that other family members may also have evolved such a circuit to limit cytotoxic IE proteins and that this circuitry could represent a broad-spectrum antiviral target.

## Data Availability Statement

The datasets generated for this study are available on request to the corresponding author.

## Author Contributions

LW conceived and designed the study. SC and RE designed and performed the experiments. SC and LW wrote the paper. All authors contributed to the article and approved the submitted version.

## Conflict of Interest

The authors declare that the research was conducted in the absence of any commercial or financial relationships that could be construed as a potential conflict of interest.

## Supplementary Material

Supplementary Movie Sixteen hour time-lapse microscopy movie of synchronized HSV-1-ICP4-YFP (strain 17syn+, MOI = 1.0) infection of ARPE-19 cells in G0.
